# The Global Measles and Rubella Laboratory Network Supports High-Quality Surveillance

**DOI:** 10.3390/vaccines12080946

**Published:** 2024-08-22

**Authors:** Paul A. Rota, Roger Evans, Myriam Corinne Ben Mamou, Gloria Rey-Benito, Lucky Sangal, Annick Dosseh, Amany Ghoniem, Charles R. Byabamazima, Maurice Demanou, Raydel Anderson, Gimin Kim, Bettina Bankamp, R. Suzanne Beard, Stephen N. Crooke, Sumathi Ramachandran, Ana Penedos, Vicki Stambos, Suellen Nicholson, David Featherstone, Mick N. Mulders

**Affiliations:** 1 Centers for Disease Control and Prevention, Atlanta, GA 30329, USA; par1@cdc.gov (P.A.R.); ofi6@cdc.gov (G.K.); bfb9@cdc.gov (B.B.); zho3@cdc.gov (R.S.B.); qjf9@cdc.gov (S.N.C.); dcq6@cdc.gov (S.R.); 2 World Health Organization Western Pacific Regional Office, Manila 1000, Philippines; evansr@who.int; 3 World Health Organization European Regional Office, 2100 Copenhagen, Denmark; benmamoum@who.int; 4 Pan American Health Organization, Washington, DC 20037, USA; reyglori@paho.org; 5 World Health Organization Southeast Asia Regional Office, Delhi 110002, India; sangallu@who.int; 6 World Health Organization African Regional Office, Brazzaville P.O. Box 06, Congo; dosseha@who.int (A.D.); byabamazimac@who.int (C.R.B.); demanoum@who.int (M.D.); 7 World Health Organization Eastern Mediterranean Regional Office, Cairo 11371, Egypt; ghoniema@who.int; 8 United Kingdom Health Security Agency, London NW9 5EQ, UK; ana.penedos@ukhsa.gov.uk; 9 Victorian Infectious Diseases Reference Laboratory, The Royal Melbourne Hospital at the Peter Doherty Institute for Infection and Immunity, Melbourne 3000, Australia; vicki.stambos@mh.org.au (V.S.); suellen.nicholson@mh.org.au (S.N.); 10 Consultant Scientists Ltd., Hastings 4122, New Zealand; featherstoned@gmail.com; 11 World Health Organization, 1211 Genève, Switzerland

**Keywords:** measles-rubella laboratory, diagnostics, network, serology, PCR, WHO

## Abstract

With 762 laboratories, the Global Measles and Rubella Laboratory Network (GMRLN) is the largest laboratory network coordinated by the World Health Organization (WHO). Like the Global Polio Laboratory Network, the GMRLN has multiple tiers, including global specialized laboratories, regional reference laboratories, national laboratories, and, in some countries, subnational laboratories. Regional networks are supervised by regional laboratory coordinators reporting to a global coordinator at WHO headquarters. Laboratories in the GMRLN have strong links to national disease control and vaccination programs. The GMRLN’s goal is to support member states in obtaining timely, complete, and reliable laboratory-based surveillance data for measles and rubella as part of the strategy for achieving measles and rubella elimination. Surveillance data are reported to the national program and are included in annual reports on the status of measles and rubella elimination to national verification committees for review by regional verification commissions. Quality within the GMRLN is ensured by monitoring performance through external quality assurance programs, confirmatory and quality control testing, accreditation, and coordination of corrective action and training where needed. The overall performance of the laboratories has remained high over the years despite many challenges, particularly the COVID-19 pandemic. The GMRLN is well-positioned to support high-quality laboratory-based surveillance for measles and rubella and to transition to supporting laboratory testing for other pathogens, including vaccine-preventable diseases.

## 1. Introduction to GMRLN

The World Health Organization (WHO) Global Measles and Rubella Laboratory Network (GMRLN) was developed using the model of the Global Polio Laboratory Network (GPLN) and the Measles Laboratory Network for the Region of the Americas as its foundation. In 1994, the Region of the Americas was certified as polio-free and, by the latter half of the 1990s, was making progress toward measles elimination. Other WHO regions had also made progress towards achieving polio-free status and were successively implementing a phased approach from control to outbreak prevention to measles elimination phases. By 1998, 115 countries had a measles elimination goal. The laboratory-based surveillance strategies for the first two phases were primarily testing a limited number of cases to confirm outbreaks and suspected spread cases. In the control and outbreak prevention phases, case confirmation was based on the detection of measles- and rubella-specific immunoglobulin M (IgM) in serum samples, though several laboratories were attempting to isolate the virus for genetic characterization. Once countries reached the elimination phase, with an incidence rate of <1 case/100,000 population, laboratories were expected to test all suspected cases and investigate virus transmission patterns to monitor interruption of endemic viral transmission. Specimens testing negative for measles IgM were tested for evidence of rubella IgM.

The GMRLN was built gradually as resources allowed, progress towards disease control was achieved, and elimination goals were set for measles and, later, for rubella. Although the GPLN was used as a model, many more laboratories were needed for the GMRLN, with almost all countries planning to have a measles-rubella laboratory. For the GMRLN, three tiers of laboratories were established. National laboratories (NLs), as nominated by member states, have a close link to national immunization and surveillance programs. Regional reference laboratories (RRLs) were designated from those laboratories demonstrating molecular testing capacity, a strong environment of quality control (QC) and quality assurance (QA), and the capacity to support NLs in their region. Finally, global specialized laboratories (GSLs) with strong technical expertise were established to support the development of protocols, establish QA and QC programs, and provide training and capacity strengthening for network laboratories at all levels. In some large countries, sub-national laboratories (SNLs) have been added to the network to share the burden of work. Financial resources were primarily from the US Centers for Disease Control and Prevention (US-CDC), and technical support was provided by WHO, US-CDC, the United Kingdom Health Security Agency (UKHSA), the Victorian Infectious Diseases Reference Laboratory (VIDRL) in Australia, the National Microbiology Laboratory, at the Public Health Agency of Canada, and the National Institute of Infectious Diseases, Japan and RRLs.

Standardization, quality assurance, and timely and accurate reporting were the foundation of the GMRLN. An evaluation of commercially available IgM assays was conducted [1], a laboratory manual was developed and printed in English, French, Chinese, Russian, and Spanish, and annual global serology proficiency and accreditation programs were established in 1999–2000. The standardized WHO nomenclature to describe measles virus genetic characteristics was established at a meeting in 1998 [2]. Since 1997, training workshops covering IgM testing and reporting were held for new laboratories, and essential equipment, test kits, and reagents were provided according to the resource needs of each laboratory. On-site accreditation reviews by laboratory experts enabled the comprehensive, standardized assessment of a laboratory’s layout, function, staffing, and interaction with surveillance programs, as well as monitoring performance over the past 12 months. The first GMRLN meeting was held in 2001 to obtain consensus on the development of the laboratory network, strengthen the integration of rubella testing, and develop a plan for improving genetic surveillance and the investigation of new technologies. At the time of the first global meeting, the GMRLN consisted of 3 GSLs, 7 RRLs, and 78 NLs (n = 88). However, the GMRLN expanded rapidly, and by 2012, there were almost 700 laboratories in the network. All countries had access to proficient laboratories or were served by one. In addition, routine testing for measles and rubella IgM exceeded 200,000 tests annually; 10,000 measles and rubella sequences had been submitted to the global databases, and annual proficiency testing, and accreditation programs were in place. The GMRLN continues to expand and support elimination quality surveillance for measles and rubella worldwide.

## 2. Structure and Governance

The GMRLN’s goal is to support member states in obtaining timely, complete, and reliable laboratory-based surveillance data for measles and rubella [3]. Surveillance data are reported to the national program and are included in reports to national verification committees (NVC). Quality is ensured by monitoring performance through external quality assurance programs, confirmatory and specialized testing, and accreditation. GSLs and RRLs provide technical assistance to NLs and SNLs for laboratory testing and provide training to expand testing capacity and maintain a stable, competent workforce. GSLs and RRLs also perform specialized and confirmatory testing, develop new assays, and maintain external quality control programs for serologic and molecular testing. It is important to note that some GSLs and RRLs also serve as NLs to support surveillance in their country. The US-CDC supports laboratories in priority countries with laboratory reagents and diagnostic kits through the International Reagent Resource (IRR) program, and the UKHSA maintains the WHO global nucleotide surveillance databases for measles and rubella, Measles Virus Nucleotide Surveillance (MeaNS) and Rubella Virus Nucleotide Surveillance (RubeNS), respectively [4].

**Figure 1:**
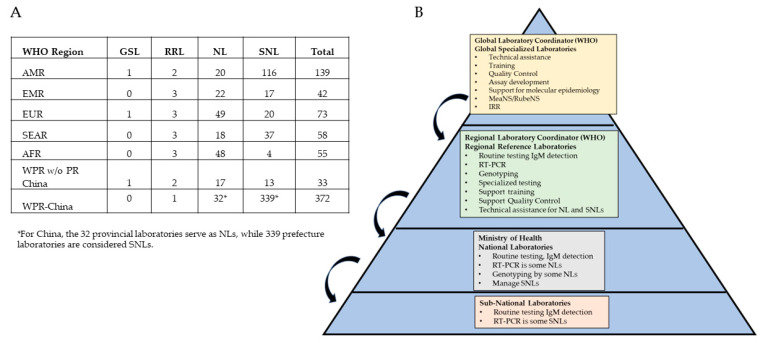
Structure and governance of GMRLN. Panel (**A**) shows the number of GSLs, RRLs, NLs, and SNLs by WHO region in 2024. Panel (**B**) shows the tiered structure of GMRLN with a summary of the major functions at each level. Curved lines indicate technical assistance and supervision.

The GMRLN is managed by a global laboratory coordinator (GLC) at WHO headquarters in Geneva. Each WHO region has one or more regional laboratory coordinators (RLC) who manage regional activities. The GLC and RLCs, along with staff of the GSLs and RRLs, form the governance structure for GMRLN (Figure 1). In addition, GMRLN has established working groups (WG) to monitor and plan specific activities and develop new approaches. The GMRLN Strategy WG comprises external, non-WHO advisers who are experts in measles and rubella within the context of the global laboratory network. The Strategy WG provides advice and guidance on testing strategies and the introduction of new technologies within the network. The Strategy WG provides input on how to address the demands for laboratory-based surveillance from the global measles and rubella program, and especially regional programs to verify the elimination of measles and rubella. Additional working groups include Next-Generation, Extended Window, and Whole-Genome (NEW) Sequencing, Rapid Diagnostic Tests (RDT), Measles and Rubella Nomenclature, IgM Test Kit Evaluation, Accreditation Checklist, Laboratory Manual, and Seroprevalence. The activities of these WGs will be described in the sections that follow.

The GMRLN established the MeaNS/RubeNS Steering Committee (SC) as an oversight board to define permissions, access, activities, roles, and responsibilities of database users. The SC also recommends modifications to the website to improve functionality. Every user must agree to the terms of the System Access Agreement and have passed the molecular external quality assurance (mEQA) that is administered by the US-CDC. The website for the databases is administered by the UKHSA and is used exclusively by GMRLN laboratories. Requests for access by non-GMRLN institutions will be considered by the SC.

Two GMRLN meetings take place each year. The winter meeting brings together the GLC, RLCs, GSLs, and other experts to provide a forum to discuss the status of the network and develop an annual work plan. The larger GMRLN meeting occurs later in the year and includes representatives from all GSLs and RRLs, as well as key NLs. At both meetings, the various working groups provide updates on progress.

## 3. Structure of Regional Networks

**Figure 2:**
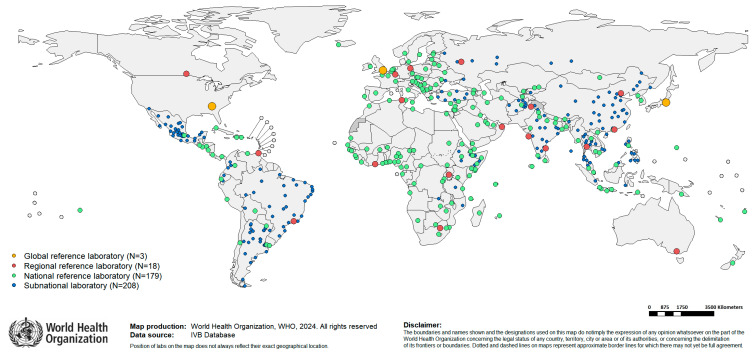
Map of GMRLN. Yellow circles indicate the locations of GSLs. Red circles indicate RRLs. Light blue circles indicate NLs. Blue circles indicate SNLs.

The GMRLN regional networks align with established WHO regions, the African Region (AFR), the Region of the Americas (AMR), the Eastern Mediterranean Region (EMR), the European Region (EUR), the South-East Asia Region (SEAR), and the Western Pacific Region (WPR). The AFR is divided into three sub-regions: Central Africa, Western Africa, and Eastern and Southern Africa. The number of RRLs in each region varies, and there is at least one NL in most member states (Figure 1 and Figure 2). Many countries have a network of SNLs. All laboratories perform serologic testing, and a growing number of laboratories have molecular testing capacity (Table 1).

In the AFR, there are three RRLs located in Uganda, South Africa, and Côte d’Ivoire. There are a total of 48 NLs including those in Algeria, Angola, Benin, Botswana, Burkina Faso, Burundi, Cameroon, Central African Republic, Chad, Comoros, Republic of Congo, Democratic Republic of Congo (DRC), Eritrea, Ethiopia, Eswatini, Equatorial Guinea, Gabon, Gambia, Ghana, Guinea, Guinea-Bissau, Kenya, Lesotho, Liberia, Madagascar, Malawi, Mali, Mauritius, Mauritania, Mozambique, Namibia, Niger, Nigeria (7), Rwanda, Seychelles, Senegal, Sierra Leone, South Africa, South Sudan, Tanzania, Togo, Zambia, and Zimbabwe. There are four SNLs in AFR, including one in DRC, two in Ethiopia and one in Mozambique.

In the AMR, there is 1 GSL/RRL, the US-CDC, 2 RRLs located in Brazil and Canada, and 20 NLs including Argentina, Bolivia, Chile, Colombia, Costa Rica, Cuba, Dominican Republic, Ecuador, El Salvador, Guatemala, Haiti, Honduras, Mexico, Nicaragua, Panama, Paraguay, Peru, Uruguay, and Venezuela. The laboratory in Trinidad and Tobago (Caribbean Public Health Agency) functions as a sub-regional reference laboratory for the Caribbean. In addition to WHO-accredited laboratories, some countries independently manage a network of SNLs, including 27 in Argentina, 27 in Brazil, 26 in Canada, 3 in Colombia, 2 in Ecuador, and 31 in Mexico. In the US, the US-CDC supports four vaccine-preventable disease reference centers [5], which provide laboratory testing support to multiple state health laboratories and departments.

In the EMR, there are two RRLs located in Oman and Tunisia, plus a sub-regional reference laboratory located in Pakistan. There are 22 NLs in total in Iran, Iraq, Egypt, Saudi Arabia, Afghanistan, Bahrain, Djibouti, Kuwait, Lebanon, Libya, Morocco, Palestine, Qatar, Somalia, Syria, United Arab Emirates, Tunisia, and 2 in Yemen. There are a few countries with SNL networks, including Afghanistan with 9 and Somalia with 11.

In the EUR, there is one GSL, the UKHSA, and three RRLs located in Luxembourg, Germany, and the Russian Federation. The region includes 49 NLs located in Albania, Armenia, Austria, Azerbaijan, Belarus, Belgium, Bulgaria, Croatia, Cyprus, Czech Republic, Denmark, Estonia, Finland, Georgia, Greece, Hungary, Iceland, Ireland, Israel, Italy, Kazakhstan, Kyrgyzstan, Latvia, Lithuania, Malta, Moldova, Montenegro, North Macedonia, Norway, Poland, Portugal, Romania, Serbia, Slovakia, Slovenia, Spain, Sweden, Switzerland, Tajikistan, Turkey, Turkmenistan, Ukraine, Uzbekistan, 2 in France, 2 in the Netherlands, and 2 in Bosnia and Herzegovina. The EUR region also has countries with SNLs that are part of the GMRLN, including 1 in Kyrgyzstan, 1 in Ukraine, 7 in Turkey, and 10 in the Russian Federation. Three countries (Italy, Kazakhstan, and Ukraine) independently coordinate their own SNL networks, and the NL from four additional countries have collaborating laboratories under their supervision (Finland, Norway, Romania, and Sweden).

In the SEAR, there are three RRLs, one in Thailand and two in India. There is a total of 19 NLs, including 7 in India, 4 in Indonesia, and 1 each in Bangladesh, Bhutan, DPR Korea, Maldives, Myanmar, Nepal, Sri Lanka, and Timor-Leste. The SNL network in SEAR consists of 18 in India, 4 in Indonesia, 1 in Nepal, and 13 in Thailand.

In the WPR, there is 1 GSL, the National Institute for Infectious Disease (NIID), located in Japan, 3 RRLs located in China, Hong Kong, and Australia, and 17 NLs, including 2 in Vietnam and 1 each in Brunei Darussalam, Cambodia, Fiji, French Polynesia, Guam, Lao PDR, Macau, Malaysia, Mongolia, New Caledonia, New Zealand, Papua New Guinea, Philippines, Republic of Korea, and Singapore. There are 13 accredited SNLs in the region: seven in the Philippines, two in Vietnam, and four in Malaysia. China established 32 WHO-accredited provincial and 339 prefectural laboratories accredited by China CDC. The GSL in Japan also independently coordinates its own SNL network.

## 4. Testing and Quality Control in GMRLN

The GMRLN supports measles and rubella surveillance by performing laboratory testing for confirmation of infection and conducting genetic characterization of circulating viruses. All the 762 laboratories perform serologic confirmation for measles and rubella infection. Serologic testing is primarily the detection of measles- and rubella-specific IgM in serum samples. GMRLN laboratories use commercial kits for IgM detection, and the quality of the kits is monitored by the GMRLN. In 2023, the GMRLN performed over 600,000 serologic tests, a significant increase in testing from 2022 (Table 2). While IgM detection is still considered the standard test for case confirmation, especially in endemic countries, many countries have included the detection of viral RNA by reverse transcription polymerase chain reaction (RT-PCR) in the testing algorithm. As countries move towards the elimination of measles and rubella, molecular testing will play an increasingly important role.

The GMRLN is responsible for the genetic characterization of wild-type measles and rubella viruses. Laboratories obtain sequence information by amplification of sequencing templates from clinical samples. The current standard protocols call for sequencing either the 450 nucleotides coding for the COOH terminal 150 amino acid region of the measles nucleoprotein gene (N-450) or 739 nucleotides from the rubella E1 gene (E1-739). Sanger sequencing methods are widely used in the GMRLN. Sequence data are submitted to the WHO global nucleotide surveillance databases, MeaNS and RubeNS. It should be noted that sequence analysis is required to distinguish between a measles vaccine reaction and infection with a wild-type virus, though vaccine-specific RT-PCR assays are being introduced in some countries [6]. The GMRLN is developing methods and quality control standards for next-generation sequencing (NGS), which will allow laboratories to obtain whole genome sequences (WGS).

In addition to the standard testing methods, GSLs and RRLs perform referral testing to help with case confirmation and case classification. Testing for immunoglobulin G (IgG) avidity is usually performed in an enzyme-linked immune assay format. High avidity IgG indicates past infection or previous vaccination with measles- or rubella-containing vaccine, while low avidity IgG is consistent with recent vaccination or infection. Avidity testing has been useful for ruling out rubella infections when IgM testing gives false positive results and for distinguishing between primary and secondary vaccine failure in measles breakthrough cases [7].

The GMRLN supports the evaluation of population immunity through serosurveys. Until recently, the most common method was enzyme immunoassay (EIA) to detect measles and rubella IgG. Only a few specialized laboratories can perform virus neutralization assays, which are the most sensitive assays for measuring virus-specific IgG concentrations. More recently, multiplexed bead assays (MBAs) have been employed to measure IgG for measles and rubella [8]. The MBA has a sensitivity equivalent to virus neutralization, is amenable to a high throughput format, and can be performed with a small amount of serum. Simultaneous testing for measles and rubella IgG saves time and resources.

As countries move towards the elimination of measles and rubella, an assessment of the quality of surveillance activities is necessary to demonstrate adequate support for verification of elimination. Five of the eight core surveillance performance indicators assess the quality and timeliness of laboratory testing and should be reported by countries to WHO regional offices. These include a proportion of countries reporting to their WHO regional office on time (target: 100%), reporting discarded non-measles non-rubella cases at the national level (target: ≥2 cases per 100,000 population per year), the proportion of suspected cases with adequate specimens for detecting acute measles or rubella infection collected and tested in a proficient laboratory (target: ≥80%), the proportion of laboratory-confirmed chains of transmission with samples adequate for detecting measles or rubella virus collected and tested in an accredited laboratory (target: ≥80%), and proportion of results reported by the laboratory within 4 days of specimen receipt (target: ≥80%). Satisfactory performance by the GMRLN in each country is a prerequisite for verification of elimination.

Quality control is the hallmark of the GMRLN. It is important that all laboratories provide accurate and consistent results to national immunization programs. Quality is improved by sharing protocols for validated methods within the network. The GMRLN has developed a detailed laboratory manual containing chapters on laboratory testing and detailed, regularly updated protocols for standard tests. The latest version of the manual was published in 2018 [9].

The GMRLN laboratories are required to participate in annual proficiency testing (PT) for measles and rubella IgM detection. The serologic PT panels for the NLs are provided by the VIDRL in Melbourne, Australia. As of 2023, there were 144 laboratories participating in the PT program. During the 2023 round of PT, 97% of laboratories submitting measles results and 98% of laboratories submitting rubella results obtained a passing score. As of May 2024, 223 laboratories submitted results for the 2023 PT program; there are additional laboratories still to submit results for this panel. For 2022, 245 laboratories participated in the PT program, and 99% of laboratories submitting measles results and 99% of laboratories submitting rubella results obtained a passing score. For those member states that have large SNL networks, the NLs are responsible for administering country-specific serology PT. The VIDRL provides training workshops for preparing and evaluating PT panels.

To fulfill requirements for WHO accreditation, in addition to passing the PT, NLs need to send 10% of tested specimens or at least 50 specimens annually to the RRL for confirmatory testing. There must be at least 90% concurrence between the results of the NL and the RRL for the NL to obtain full accreditation status. For any laboratory not reaching the minimum concurrence or not submitting samples for QC, its accreditation standing may reflect a provisional status until it returns to compliance.

The purpose of the mEQA program of the GMRLN is to assess the ability of network laboratories to perform molecular testing. Molecular testing includes assays to detect viral RNA, i.e., real-time RT-PCR (rRT-PCR) or endpoint RT-PCR for rubella, as well as assays used for routine genotyping (measles N-450 and rubella E1-739). Additionally, all GMRLN laboratories that report sequences to the global databases, MeaNS and RubeNS, are required to pass the sequencing portion of the mEQA. In 2022, 119 NLs participated in the mEQA for measles, and 108 participated for rubella. Of these, 95% and 96% passed measles and rubella proficiency testing, respectively. Laboratories that fail any portion of the mEQA must pass a repeat test before they can submit sequence data to the global databases. Countries with a large SNL network are responsible for country-specific mEQA. US-CDC provides training workshops to NLs preparing mEQA panels to ensure consistency with proficiency testing.

WHO accredits all GMRLN laboratories based on a standard checklist of requirements. Accreditation is achieved by onsite visits as well as desk reviews. New laboratories will receive an initial onsite accreditation followed by onsite visits every 2–5 years, depending on the region. Most laboratories are required to complete the accreditation checklist for desk review annually. The Accreditation WG completed a revised version of the checklist in 2024.

## 5. Introduction of New Technologies

The GMRLN has designated a working group focusing on developing and deploying rapid diagnostic tests (RDTs) for measles and rubella, including a framework for introducing RDTs to national surveillance programs [10]. RDTs were a game changer during the COVID-19 pandemic, and RDTs are expected to impact the timeliness and sensitivity of measles and rubella surveillance. One challenge considered by the RDT WG is the reporting of negative test results needed to evaluate the performance of national surveillance programs. Along with the WHO, various partners, including the Bill and Melinda Gates Foundation, Gavi the Vaccine Alliance, US-CDC, UKHSA, UNICEF, and FIND, have made great efforts to introduce newly developed measles and rubella testing formats (e.g., RDTs) to the GMRLN. Besides supporting the development and production of RDTs, the WG is developing guidance for the introduction of RDTs. 

Three additional WGs with overlapping roles are focusing on improving serologic testing. One WG is involved in the validation of enzyme-linked immune assays. This WG developed a target product profile to define the minimum user requirements of measles and rubella EIA and RDT assays target product profile [11]. The target product profile allows commercial manufacturers of EIA and RDT assays to submit an expression of interest in participating in the evaluation study, which will be advertised in the United Nations Marketplace. One of the RRLs, the Public Health Agency of Canada, has agreed to conduct the laboratory evaluation of the assays and provide a report, which will be evaluated by the GMRLN and made available on the WHO website. Another WG is investigating the reasons for discrepant IgM test results. Recommendations from this WG will be available in the next version of the laboratory manual. A third WG is involved in the development and use of the MBA [8], also called multiplex immunoassay [12], to facilitate seroprevalence studies.

## 6. Molecular Epidemiology

Genetic characterization of wild-type measles and rubella viruses is a critical component of the global surveillance program. Data from virologic surveillance can map viral transmission pathways, allowing linkage of cases and outbreaks and identification of possible sources of importation. Characterization of the genetic properties of circulating wild-type viruses helps monitor progress toward elimination goals. An essential element of verification of measles and rubella elimination is documentation of ≥12 months with no continuous circulation of the virus in a country with a well-performing surveillance system; verification of elimination is achieved after ≥36 months of uninterrupted endemic viral transmission. Virologic surveillance data provided in each country’s annual NVC report to the regional verification commission constitutes a critical line of evidence supporting the verification criteria. The GMRLN plays an essential role in this process by generating high-quality sequence data. At the country level, understanding virus transmission allows the identification of immunization gaps and the tailoring of strategies for the intervention [13]. 

**Figure 3:**
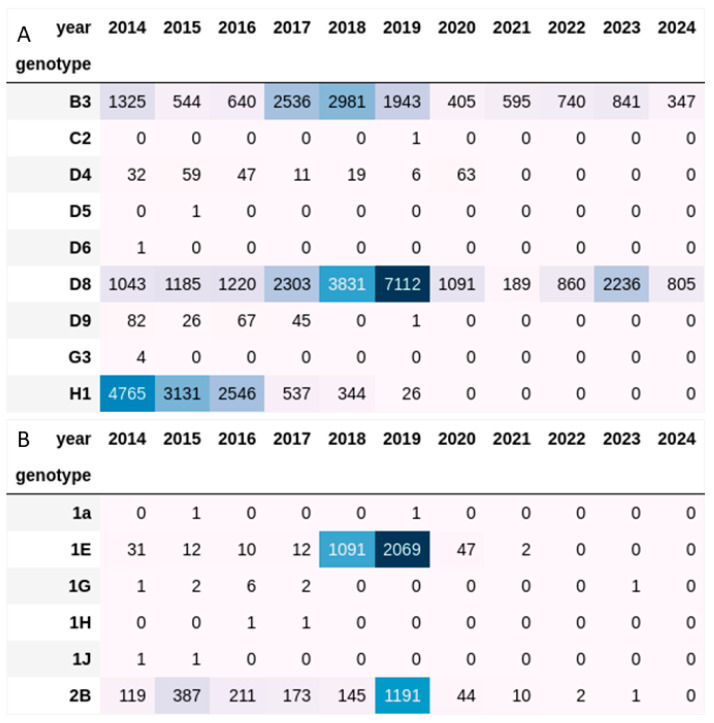
Heatmap showing the number of measles (panel (**A**)) and rubella (panel (**B**)) sequences submitted to MeaNS and RubeNS by genotype for the year 2014 through March 2024.

While WHO recognizes 24 genotypes of measles, the usefulness of standard genotyping for monitoring measles transmission is now limited because only two genotypes, B3 and D8, have been detected since 2021 (Figure 3A). Genetic diversity within a genotype is monitored by using a distinct sequence identifier (DSId) for each unique N-450 sequence. The GMRLN has provided guidance for countries on how to present data from measles N-450 sequences in reports to NVCs [13]. However, with the decrease in genetic diversity, the utility of N-450 sequence analysis to resolve measles transmission pathways is reduced. Increasing the amount of sequence data obtained from each case could improve the resolution of sequence analysis. The GMRLN supports efforts to sequence larger parts of the genome, including using NGS methods for obtaining WGS [14]. The NEW working group evaluated various methods developed by network laboratories for their applicability to virologic surveillance. Guidance for the introduction of new sequencing methods has been published by the NEW working group in the laboratory manual [9]. The NEW working group is involved in defining and developing methods for quality assurance of next-generation sequencing. 

Rubella genotypes reported to RubeNS in the last 10 years were genotype 1E (58%) and genotype 2B (42%) (Figure 3B). However, most of the sequences were from outbreaks in only two countries, China, and Japan, highlighting the need to enhance global virologic surveillance for rubella [15]. Analysis of rubella genotypes helped support the verification of the elimination of rubella in the Americas. A nomenclature has been proposed to describe the intergenotypic diversity of rubella viruses [16]. 

The Measles and Rubella Nomenclature WG with the GMRLN is analyzing the sequences submitted in MeaNS and RubeNS to define lineages of measles and rubella viruses. This will support a more precise classification of the genetic variants of measles and rubella and improve the ability of sequence data to monitor transmission chains. 

## 7. Training

To maintain high-quality work, there is a continual need for training within the GMRLN. Training is provided in multiple formats, hands-on, conducted on-site, or virtually. Most of the recent training activities have focused on methods for molecular biology since serologic testing is well established within the GMRLN; however, training for IgM testing and IgG avidity testing are also offered. Since 2022, twelve regional workshops have been conducted in AFR, AMR, EMR, and EUR, with a focus on molecular and serologic methods (Table 3).

In these workshops, US-CDC provided training to 230 participants from 60 countries through 18 separate workshops (Table 4). Each training workshop serves a unique purpose, with agendas based on regional requirements. On-site workshops involve hands-on training in technical and analytical procedures for rRT-PCR, conventional RT-PCR, and Sanger sequencing associated with the detection and genotyping of measles and rubella viruses. Laboratory exercises are followed by hands-on sequence analysis using RECall^®^ software (version 2.34) and phylogenetic analysis using MEGA^®^ software (version 11). The use of MeaNS and RubeNS databases to track circulating virus lineages and nomenclature for measles and rubella was implemented in the training. Quality control and troubleshooting were also discussed. Follow-up assessments were always conducted to ensure the acceptability of results from the trained laboratories. Participants were provided with practice panels to submit to the CDC for evaluation.

To build regional training capacity, train the trainers (TTT) workshops have been organized. In addition, the US-CDC has initiated a program to train regional focal points for laboratory training. This TTT is designed to harmonize the approaches to and methods for training and to build regional training capacity. Selected GMRLN scientists spend eight to ten weeks at the US-CDC measles and rubella laboratories in Atlanta, learning techniques, troubleshooting, and analysis, as well as giving presentations. A teach-back session for each method with a hands-on teaching component is part of the TTT workshops to ensure that the knowledge transfer is successful. Ultimately, this TTT program will lead to sustained training capacity in the regions.

## 8. Achievements and Challenges

Overall, the performance of the GMRLN has remained high despite many challenges. While the most significant challenge was the disruption caused by the COVID-19 pandemic, other challenges include limited financial resources for testing and regional coordination, staff turnover in national laboratories, updating molecular epidemiology, integration of new technologies, expansion of the network, lack of serum samples for the serologic proficient testing program, and developing testing strategies for pre- and post-elimination settings. Laboratory surveillance indicators in all regions remain high, although many countries struggle to meet the discard rate of 2/100,000 population.

Now that global travel has returned to pre-pandemic levels, the GMRLN has been able to address staff turnover by providing training opportunities for new staff. These trainings help build a stable and competent workforce, increase capacity, and introduce new or modified testing methods. To address the training needs for this increasing network, regional training capacity will need improvement with the assignment of regional training focal points and continuation of the TTT programs.

The Measles and Rubella Strategic Framework 2021–2030 [17], developed as part of the Immunization Agenda 2030 (IA2030) [18], reinforces measles and rubella elimination as a critical goal. In addition, IA2030 calls for using measles as a tracer to identify weaknesses in immunization programs. Therefore, strong laboratory-based surveillance will be a critical factor in monitoring programmatic achievements. Many countries are now expanding their domestic laboratory capacity by enrolling new laboratories, so the GMRLN keeps expanding. Robust serology and molecular EQA programs monitor the performance of the network laboratories. With that expansion also comes a greater need for EQA programs, creating an increased demand for WHO-coordinated EQA programs. Several countries have established EQA programs for SNLs, and efforts are underway to train national laboratories in larger countries with an SNL network to conduct their own EQA programs. The RLCs have worked with VIRDL to identify sources of serum samples for the serologic proficiency testing program.

The WHO remains committed to continue coordinating the GMRLN through its GLC and RLCs by organizing regular meetings and workshops. Resources are needed to maintain regional coordination for the GMRLN. A game-changer for the network has been the IRR, which took over the provision of proficiency test panels, diagnostic kits, and reagents from the WHO, significantly increasing delivery speed in most countries. RLCs work with the CDC to manage the distribution of materials and monitor inventories to minimize stockouts.

During the pandemic, a major investment was made to introduce molecular testing capacity for SARS-CoV-2 on a wide scale. This created an opportunity to introduce routine molecular testing for other pathogens. Many of the laboratory staff in measles and rubella laboratories have been trained in molecular diagnostics, and with the widespread availability of equipment, many of these laboratories are now adding rRT-PCR for measles and rubella to their portfolio as an added tool to confirm suspect cases. The pandemic also made sequencing accessible to many more laboratories. The GMRLN must make sure network laboratories have the capacity to use rRT-PCR as part of the case confirmation algorithm and generate high-quality sequence data to monitor viral transmission pathways. New, validated protocols and subsequent training will be needed. The GMRLN will continue its reliance on the MeaNS and RubeNS databases, particularly now that many laboratories are transitioning to NGS. Viral genetic diversity has decreased in recent years, creating a need to better describe genetic variability with measles and rubella genotypes. Therefore, there is a critical need to expand capacity for sequencing and to develop an updated nomenclature to describe intragenotype variation. 

With an ever-increasing laboratory network, one of the biggest challenges for the GMRLN is its need to diversify its source of funding to maintain and expand its capacity and capability. The US-CDC has been a generous provider of resources to the GMRLN since its inception, not only in funds but also in technical assistance, staff training, and the provision of quality laboratory management programs. This support has been crucial to the GMRLN’s development. There has also been a strong reliance on the GPLN since the initiation of the GMRLN. Many investments made into building infrastructure and laboratory capacity in the GPLN have been beneficial to other vaccine-preventable disease laboratory networks such as GMRLN, Yellow Fever, and rotavirus [19]. This synergy has been very beneficial and a cost-effective use of donor support. With polio eradication approaching, a process of transition has been initiated as described in the Strategic Action Plan on Polio Transition [20]. This transition creates a need to integrate activities between polio and other programs, including laboratory networks. A revised action plan is under development to describe the opportunities during transition. The WHO has developed a global strategy for comprehensive vaccine-preventable disease (VPD) surveillance [21,22]. This strategy promotes the development of comprehensive high-functioning surveillance systems that encompass all VPD threats faced by a country in all areas and populations, wherever possible, taking advantage of the shared infrastructure for components of surveillance such as data management and laboratory systems. 

One of the five main objectives of the global strategy is to strengthen and expand public health laboratory networks. The GMRLN has an excellent capacity to conduct basic and advanced laboratory testing, as shown by the outcomes of the accreditation and quality control programs. Therefore, the GMRLN is well-positioned to support high-quality laboratory-based surveillance for measles and rubella and to transition to supporting laboratory testing for other pathogens, including vaccine-preventable diseases. Expansion of the GMRLN will be based on the guiding principles of high-quality testing, outstanding quality control, and integration with national and regional disease control plans.

## Figures and Tables

**Table 1:** Number of laboratories in the GMRLN performing molecular testing in each WHO region.

WHO Region	NL Performing Virus Detection	Sequencing Labs
AMRO	22	9
EMRO	8	7
EURO	43	32
SEARO	18	5 *
AFRO	10	6
WPRO	13	45

* 5 labs perform sequencing, but 35 perform sequence analysis.

**Table 2:** Workload of the GMRLN in 2022–2023.

GMRLN Testing by Year	Specimens Received	Specimens Tested
2023	402,883	Measles 333,835
		Rubella 286,780
		Total tests 620,615
2022	272,990	Measles 217,179
		Rubella 209,992
		Total tests 427,171

**Table 3:** Regional training activities by the GMRLN in 2022–2023.

WHO Region	Training	Date	Participants	Lead
AFR	Molecular	November 2022	Eritrea, Kenya, Mauritius, Mozambique, Seychelles, South Africa, Zambia	US-CDC, RRL Uganda Virus Research Institute
EUR	Serology webinar	May 2023	Armenia, Kazakhstan, Kyrgyzstan, Turkmenistan	RRL Moscow
EUR	GT/sequencing	May 2023	Serbia	RRL Luxembourg
AFR	Serology	September 2023	Equatorial Guinea	RRL Pasteur Institute Cote d’Ivoire
EUR	Advanced training in serology/molecular	September 2023	Moscow RRL	RRL Luxembourg
EUR	Serology/rRT-PCR/sequence analysis/MeaNS	October 2023	Entire network (group-specific)	London and US-CDC, RRLs: Luxembourg, Berlin, and Moscow
EUR	Measles rRT-PCR hands-on training	November–December 2023	Kyrgyzstan NL and SNL Uzbekistan Tajikistan	RRL Moscow
EMR	ELISA for measles and rubella, measles rRT-PCR	September 2023	CPHL Kabul	NIH Islamabad
EMR	Serological detection of measles and rubella virus	November 2023	Somalia	RRL Tunisia, Pasteur Institute
EMR	MR outbreak simulation exercise	November 2023	Egypt	Egypt
EMR	Workshop on measles and rubella verification of elimination	February 2023	Kuwait, Qatar, Saudi Arabia, UAE	United Arab Emirates
AMR	Measles and rubella rRT-PCR	November 2023	Nicaragua	AMR

**Table 4:** Training activities provided by the US CDC and WHO’s Regional Offices in 2023.

Training Location	Training	Date	Participants
Webinar	Webinar on measles and rubella diagnostic real-time RT-PCR	January 2023	Angola
Webinar	Webinar on measles and rubella diagnostic real-time RT-PCR	January 2023	Armenia, Azerbaijan, Belarus, Kazakhstan, Kyrgyzstan, Moldova, Tajikistan, Turkmenistan, Uzbekistan
National Institute of Health, Bangkok, Thailand	Strengthening molecular capacity for the Global Measles and Rubella Laboratory Network in the South-East Asia Region	March 2023	Bangladesh, Bhutan, Indonesia, Thailand, Maldives, Nepal, Sri Lanka, Timor-Leste
Institute of Epidemiological Diagnosis and Reference Laboratory (InDRE), Mexico	Measles and rubella genotyping workshop	September 2023	Colombia, Mexico, Peru, Trinidad and Tobago, Uruguay
Nigeria Centre for Disease Control (NCDC), Nigeria	“Train the Trainers” serology workshop and molecular techniques for the detection of measles and rubella	October 2023	Lagos Central Public Health Laboratory and the National Reference Laboratory
Istanbul, Türkiye	Molecular techniques for the detection of measles and rubella	October 2023	European countries
Webinar	Use of Sequencher^®^ (version 5.4.6) for sequence analysis of measles and rubella	December 2023	India, Hong Kong, Thailand, Bangladesh

## Data Availability

The data presented in this study are available in this article.
